# Differential Risk: Gender and Racial Differences in the Relationship between Trauma, Discrimination, and Schizotypy

**DOI:** 10.3390/bs14050363

**Published:** 2024-04-25

**Authors:** Mahogany A. Monette, Madisen T. Russell, Danielle B. Abel, Jarrett T. Lewis, Jessica L. Mickens, Evan J. Myers, Megan M. Hricovec, David C. Cicero, J. Wolny, William P. Hetrick, Michael D. Masucci, Alex S. Cohen, Christopher J. Burgin, Thomas R. Kwapil, Kyle S. Minor

**Affiliations:** 1 Department of Psychology, Indiana University Indianapolis, Indianapolis, IN 46202, USA; madtruss@iu.edu (M.T.R.); jmickens@iu.edu (J.L.M.); evjmyers@iu.edu (E.J.M.); ksminor@iupui.edu (K.S.M.); 2 Department of Educational Psychology, University of Illinois Urbana-Champaign, Champaign, IL 61820, USA; jtlewis@illinois.edu; 3 Department of Psychology, University of North Texas, Denton, TX 76201, USAdavid.cicero@unt.edu (D.C.C.); 4 Department of Psychological and Brain Sciences, Indiana University, Bloomington, IN 47405, USA; wolny@iu.edu (J.W.);; 5 Department of Psychology, Louisiana State University, Baton Rouge, LA 70803, USA; 6 Department of Psychology, Tennessee Technological University, Cookeville, TN 38505, USA; cburgin@tntech.edu; 7 Department of Psychology, University of Illinois Urbana-Champaign, Champaign, IL 61820, USA; trkwapil@illinois.edu; 8 Department of Psychology, University of North Carolina at Greensboro, Greensboro, NC 27412, USA

**Keywords:** trauma, racial discrimination, schizotypy, disparities

## Abstract

Traumatic experiences are associated with increased experiences of positive schizotypy. This may be especially important for People of Color, who experience higher rates of trauma and racial discrimination. No study to date has examined how racial disparities in traumatic experiences may impact schizotypy. Furthermore, of the studies that have examined the relationship between trauma and schizotypy, none have examined racial discrimination as a potential moderator. The present study examined if racial discrimination moderates the relationship between trauma and multidimensional (positive, negative, and disorganized) schizotypy. In a sample of 770 college students, we conducted chi-squared analyses, analyses of variance, and stepwise regressions. We found that Black students experienced significantly higher racial discrimination and trauma than Latinx and Asian students. Furthermore, Black and Latinx students experienced significantly more multidimensional schizotypy items than Asian students. Trauma and racial discrimination explained 8 to 23% of the variance in each dimension of schizotypy. Racial discrimination did not moderate the relationships between trauma and multidimensional schizotypy. Our findings suggest that we need to examine risk factors that may prevent recovery from psychotic disorders. Additionally, disorganized schizotypy showed the most robust associations and may be a critical site of intervention.

## 1. Introduction

Psychotic disorders, marked by chronicity and functional impairment, present substantial individual difficulties [1,2,3]. Despite these challenges, several factors predict or modify the course of schizophrenia and individuals’ recovery outcomes, including not only clinical and treatment features (e.g., symptom severity, substance abuse, and duration of illness), but also demographic characteristics (e.g., age at onset and sex) and socioeconomic variables [4]. However, systematic barriers such as provider bias and racism persist and impede marginalized racial groups’ trust in the healthcare system, access to quality care, and recovery [5,6,7]. Moreover, although marginalized racial and ethnic groups experience higher rates of trauma than their White counterparts, they are also more likely to report unmet mental healthcare needs, especially following traumatic life events [8,9,10]. Given the profound associations between trauma and psychotic-like experiences, particularly for marginalized populations, these barriers are of particular importance. However, the function and pathways of these factors remain largely unexplored. 

Recognizing the challenges posed by systematic barriers requires a broader perspective of psychosis. Psychotic disorders represent the extreme end of the schizotypy continuum [11,12], involving a spectrum of clinical and subclinical manifestations of traits characterized by positive (e.g., odd thinking, unusual perceptual experiences, and paranoia), negative (e.g., alogia, avolition, and anhedonia), and disorganized (e.g., difficulties with cognition and speech) dimensions [11,13,14]. This conceptualization extends beyond traditional diagnostic boundaries, emphasizing a continuum influenced by genetic and environmental factors, allowing researchers to examine how environmental factors (e.g., racial/ethnic disparities and trauma) may increase the risk of psychopathology.

Racial and ethnic disparities in experiences of schizotypy are well documented, although it is unclear if these are due to a lack of culturally appropriate measures. Despite potential measurement insensitivity, meaningful disparities may exist. Cohen and Marino [15] found that Black and Latinx participants in the general population reported being more distressed by experiences of psychosis than Asian and White participants. Barrio and colleagues [16] found that Black participants experienced more hallucinatory behavior and suspiciousness than White participants, whereas Latino participants experienced more somatic concerns across the schizophrenia spectrum. Barrio and colleagues [16] discussed that positive symptoms may be exacerbated by racism and emotional distress, suggesting that racism is an especially plausible risk factor for elevated rates of schizotypy and psychosis among People of Color.

### 1.1. Racial Discrimination and Schizotypy

Racial discrimination is positively associated with subthreshold psychotic experiences [17,18,19,20,21,22,23,24]. Findings from two epidemiological studies in the United States of America found that increased perceived racial discrimination was associated with higher auditory and visual hallucinations and delusional ideation [22]. In a nonclinical sample of Students of Color, Anglin and colleagues [18] found that perceived racial discrimination was significantly and positively associated with attenuated positive psychotic symptoms (e.g., suspiciousness, perceptual abnormalities, unusual thinking, and cognitive disorganization). Thus, racial discrimination may be associated with subclinical positive and disorganized symptoms of psychosis among People of Color. However, there are currently no empirical publications examining the relationship of racial discrimination to negative symptoms of psychosis—a notable gap, given that all three dimensions of subclinical symptoms may precede the development of psychotic disorders [25,26]. 

### 1.2. Trauma

Traumatic experiences are also a risk factor for psychosis [27,28,29]. Among a sample of 68 patients experiencing or at risk of psychosis, Martin and colleagues [30] found that 95% reported at least one traumatic event and 65% of patients linked these events to their psychotic symptoms. In the United States, Gibson and colleagues’ [28] meta-analysis suggested that trauma increases the prevalence of positive symptoms, particularly when the traumatic event is interpersonal and perceived as intentional. Whilst there is limited literature on the relationship between trauma and negative or disorganized symptoms of psychosis, existing findings suggest that there is not a significant relationship [29,31,32,33,34]. Beards and colleagues [27] found that in 14 of the 16 studies reviewed in their meta-analysis, individuals experiencing psychosis were three times more likely to have experienced recent adverse life events, with gender appearing to be a potential moderator of this effect. Further, Mansueto and Faravelli [35] found that the relationship between recent life events and psychosis is stronger for women than men. However, this study did not include gender-minority participants (e.g., nonbinary or transgender), suggesting the need for gender-inclusive research to examine the association between trauma across the lifespan and multidimensional schizotypy. 

### 1.3. Racial Disparities in Exposure to Trauma

It is important to examine potential risk factors for experiences of post-traumatic stress disorder (PTSD) among People of Color at risk of symptoms of psychosis. Using data from the Collaborative Psychiatric Epidemiology Survey, McLaughlin and colleagues [36] found that Black participants had the highest incidence rate of PTSD (6.73%), followed by White (5.59%), Latinx (3.77%), and then Asian (1.64%) participants. There were also racial disparities in the type of traumatic exposure: Asian participants experienced more exposure to organized crime, Black participants experienced more sexual violence and other traumatic events, and Latinx participants experienced more exposure to physical violence victimization. These forms of trauma among Black, Asian, and Latinx adults may indicate distinct features among these groups, particularly given the relationship between interpersonal trauma and psychosis. 

Racial discrimination may also worsen the relationship between trauma symptoms and mental health. Kirkinis and colleagues’ systematic review [37] found that 70% of studies reviewed reported a positive relationship between racial discrimination and traumatic symptoms. Additionally, the relationship between racial discrimination and PTSD is strongest for Black people, followed by Latinx, then Asian adults [38]. Gender may also impact this relationship as Mekawi and colleagues [39] found that racial discrimination moderated the relationship between interpersonal trauma and PTSD in their sample of Black women such that the association between PTSD symptoms and interpersonal trauma was stronger at higher levels of racial discrimination. Given the associations between racial discrimination and trauma symptoms with schizotypy, exploring the potentially combined impact of these two risk factors on People of Color is crucial as the strength of this relationship may vary by racial group and prior experiences of racism.

### 1.4. Goals and Hypotheses

The present study examined the relationship between trauma and multidimensional schizotypy as well as the extent to which racial discrimination moderated this relationship in a sample of Adults of Color. Understanding gender and racial disparities in the prevalence of trauma, racial discrimination, schizotypy, and the interplay between these variables is critical for the development of culturally syntonic assessments and interventions that support recovery from serious mental illness. Our hypotheses were as follows:

(1) Black and Latinx participants have a greater likelihood of experiencing racial discrimination than Asian participants.
(2) Black and Latinx participants have a greater likelihood of experiencing trauma than Asian participants, whereas women and gender-minority participants have a greater likelihood of experiencing interpersonal trauma than men.
(3) Black and Latinx participants have a greater likelihood of experiencing positive and disorganized, but not negative, schizotypy than Asian participants.
(4) Racial discrimination is correlated with positive and disorganized, but not negative, schizotypy.
(5) Trauma is positively correlated with positive, but not negative or disorganized, schizotypy.
(6) Previous experiences of racial discrimination moderate the relationship between experiences of trauma and positive schizotypy.

## 2. Methods

### 2.1. Setting

Participants were recruited from Psychology Department subject pools at six universities in the Midwestern and Southern United States of America in the Fall of 2021 and Spring of 2022. 

### 2.2. Participants

The inclusion criteria for the study were that participants had to be at least 18 years of age and identify as Black or African American, Asian, and/or Latinx. Multiracial individuals were not included in the analyses. A total of 848 participants who met the inclusion criteria completed the study; however, 78 participants were removed due to invalid response patterns based on elevated scores on an infrequent responding measure (see Measures) or not completing measures of interest, leaving a final sample of 770 participants. The mean age of the sample was 19.2 years (SD = 2.0), participant ages ranged from 18 to 43 (see Table 1), and 73.0% of the sample identified as women. This study was approved by all universities’ Institutional Review Boards and electronic informed consent was obtained from all participants prior to enrollment. All participants received course credit for their participation in the study. 

### 2.3. Variables

Participants completed questionnaires assessing demographic characteristics, multidimensional schizotypy, trauma, racial discrimination, and infrequent responses.

Demographics. The demographic questionnaire assessed participants’ age, gender, and race and ethnicity. Gender options included no answer, cisgender man, cisgender woman, transgender, gender queer or gender nonconforming, or a gender not listed here. Due to fewer people selecting these last three items, people who experienced these categories were all relabeled as gender-minority. Participants could choose multiple options from the following racial categories: Black, Asian, and White. Additionally, participants were asked if they identified as Hispanic or Latinx. Participants who endorsed more than one race and/or ethnicity (e.g., Asian Latinx participants) were excluded from the analyses so that racial and ethnic groups could be compared.

Multidimensional Schizotypy. The Multidimensional Schizotypy Scale (MSS [40]) is a 77-item, true–false measure of positive, negative, and disorganized schizotypy. The scale development focused on retaining items that had minimal differential item functioning for race and sex. Following the authors’ recommendations, separate scores were computed for the positive, negative, and disorganized subscales. Higher scores indicated greater experience of schizotypy items. Sample items for the MSS include “I have sometimes felt that strangers were reading my mind” (positive schizotypy), “I rarely feel strong emotions even in situations in which other people usually do” (negative schizotypy), and “When people ask me a question, I often don’t understand what they are asking” (disorganized schizotypy). The MSS has demonstrated good internal consistency and test–retest reliability in adult samples [41]. Questionnaire [41], interview [42], and ambulatory assessment [43] studies support the construct validity of the MSS. Furthermore, Rbeiz et al. [44] and Li et al. [45] indicated that the predictive validity of the MSS is invariant across racial groups. 

Trauma. The Life Event Checklist for Diagnostic and Statistical Manual of Mental Disorders Fifth Edition (LEC-5 [46]; DSM-5) is a 17-item instrument assessing lifetime potentially traumatic events. Experiences include, but are not limited to, natural disasters, unwanted sexual experiences, physical assault, and assault with a weapon. For this study, we also included a supplemental item, prolonged separation from a caregiver. For each event, participants chose one of the following responses: doesn’t apply, not sure, learned about it, witnessed it, or happened to me. Item-level analyses were conducted to examine the variety and frequency of potentially traumatic events in this sample.

Participants also completed the PTSD Checklist for DSM-5 (PCL-5 [47]), a 20-item scale assessing responses to traumatic experiences in the previous week. Participants reported their distress from these responses on a five-point Likert scale: (0) “Not at all”, (1) “A little bit”, (2) “Moderately”, (3) “Quite a bit”, and (4) “Extremely”. A sample item is “In the past week, how much were you bothered by: Repeated, disturbing, and unwanted memories of the stressful experience?” Responses to all items were summed, resulting in a total score ranging from 0 to 80. The PCL-5 has established reliability and validity in adult samples [48]. Reliability in our sample was strong (alpha = 0.95).

Racial discrimination. The Major Lifetime Discrimination Scale (MLDS [49]) examines self-reported major experiences of discrimination. The survey enquires about discrimination at school, work, being hired for a job, medical settings, receiving service in a store, seeking credit or a bank loan, in the street, and from police and judicial systems. For each event, participants are asked which aspect of their identity they believed was the basis of their discriminatory experience. For this study, participants were only counted as endorsing a specific event if they attributed the discrimination to their race, ethnicity, or color. The Major Lifetime Discrimination Scale generates incidence and frequency scores. For the incidence score, all nine items are summed for a score ranging from 0 to 9. Participants report the frequency of these experiences across their lifetime from zero to three: (0) never happened; (1) once; (2) two to three times; and (3) four or more times. The frequency score is summed across all nine incidences and ranges from 0 to 27. The scale has good reliability and validity among Black and Latinx adults [50]. A subset of items was used in the 2016 National Asian American Survey and showed good validity [51]. 

Infrequency Questionnaire. Chapman and Chapman’s [52] 13-item true–false infrequency questionnaire was embedded within the MSS to screen out invalid responders from the study. Participants who experienced more than three infrequency items were excluded from the analyses.

### 2.4. Sampling and Sample Size

To determine the required sample size, we reviewed effect sizes from comparable studies of racial discrimination, trauma, and schizotypy. Our hypothesized effects had medium to large effect sizes in previous studies, including Cohen’s *f*^2^ = 0.20 [18], adjusted odds ratios of 4.59 [22] for discrimination and positive symptoms of psychosis, and small to moderate effect sizes for trauma and psychosis, with correlations ranging from 0.11 to 0.30 and a weighted odds ratio of 3.19 (95% CI: 2.15–4.75) [27]. According to Cohen [53], a minimum sample size of 547 would ensure a power of 0.80 for this range of effect sizes for regression analyses with two predictors at alpha = 0.05. We recruited a final sample of 770 participants; therefore, our analyses were adequate for the detection of small effect sizes.

### 2.5. Statistical Analyses

Analyses were conducted using International Business Machines (IBM) Statistical Package for Social Sciences version 29 (SPSS). Chi-squared tests for independence and one-way analysis of variances (ANOVAs) were used to compare demographic groups (i.e., race and ethnicity and gender) with trauma, racial discrimination, and multidimensional schizotypy. Subscale scores for positive, negative, and disorganized schizotypy were computed following Gross and colleagues’ [54] recommendations. Given the high skew in our variables of interest (trauma and racial discrimination), the variables were logarithmically transformed.

To test our hypotheses, we conducted moderation analyses, a process analysis that examines if the strength of the relationship between X and Y is dependent on the level of another variable, W. We used the PROCESS macro in SPSS for these analyses [55]. Traumatic distress was our independent variable (X), schizotypy was our outcome variable (Y), and racial discrimination was our moderating variable (W). Each model was run three separate times using positive (model 1), negative (model 2), and disorganized schizotypy (model 3) as the outcome variables. Gender (man, woman, and gender-minority) was included as a covariate in these models. To probe significant interactions, confidence intervals were generated for the indirect effect at different levels of racial discrimination at 1 standard deviation (SD) below the mean, the mean, and 1 SD above the mean.

## 3. Results

### 3.1. Descriptive Analyses

Table 1 includes descriptive data and information on our variables of interest. Participants in this sample produced PCL-5 scores ranging from 0 to 80 (M = 20.85; SD = 17.72). About one-fourth of our sample (24.4%) met the clinical cut-off for PTSD (PCL-5 score of 33 or greater). Frequency of racial discrimination responses ranged from a total score of 0 to 25 (M = 4.52; SD = 4.68). Positive schizotypy scores ranged from 0 to 26 (M = 5.59; SD = 5.30). Negative schizotypy scores ranged from 0 to 21 (M = 4.24; SD = 4.54). Disorganized schizotypy scores ranged from 0 to 25 (M = 6.32; SD = 6.90). Prior to the aggregating studies, we tested if universities differed on clinical variables. Significant differences were observed between samples for positive, negative, and disorganized schizotypy as well as trauma scores. In each instance, differences were observed between three samples in the Midwest. The pattern of differences was consistent, with those in Sample 1 demonstrating less positive, negative, and disorganized schizotypy and trauma than Samples 2 or 3 but not samples 4, 5, or 6. These differences appeared to be due to the proportion of various racial groups at each institution. There were demographic differences in trauma, racial discrimination, and multidimensional schizotypy, which are described below. 

### 3.2. Trauma

Our team observed demographic differences in traumatic events experienced, as measured by the LEC-5. A one-way ANOVA revealed that there was a statistically significant difference in types of traumas experienced between at least two groups (see Table 2 and Table 3). Regarding gender disparities (see Table 2), women and gender minorities were more likely than men to experience sexual assault (*p* < 0.01) and other unwanted sexual experiences (*p* < 0.03). Men were more likely to experience severe human suffering (*p* = 0.03), work-related accidents (*p* = 0.007), and harm they caused to someone else (*p* = 0.007) than women. Finally, gender-minority participants were significantly more likely than women to experience prolonged separation from a caregiver (*p* = 0.062). Tukey’s post hoc analyses revealed that Latinx participants were more likely to experience sexual assault than Asian participants (*p* = 0.002). Black and Latinx participants experienced more unwanted sexual experiences than Asian participants (*p* < 0.001). Black participants were more likely to experience the sudden unexpected death of someone close to them than Asian participants (*p* = 0.002). 

### 3.3. Racial Discrimination

Our team observed demographic differences in the frequency of racial discrimination. A one-way ANOVA revealed that there was a statistically significant difference in the frequency of discrimination experienced between at least two groups (see Table 4). Tukey’s post hoc analyses revealed that Black participants were more likely to experience racial discrimination at school than Latinx participants (*p* < 0.001). Black participants experienced more racial discrimination than Latinx and Asian participants getting a job (*p* < 0.001), at work (*p* < 0.001), getting housing (*p* = 0.003), getting medical care (*p* < 0.001), getting service in a store or restaurant (*p* < 0.001), getting credit or a bank loan (*p* < 0.01), in public (*p* < 0.04), and from the police or courts (*p* < 0.001). Black participants were more likely to experience the sudden unexpected death of someone close to them than Asian participants (*p* = 0.001). 

### 3.4. Multidimensional Schizotypy

Our team observed demographic differences in the frequency of multidimensional schizotypy. A one-way ANOVA revealed that there was a statistically significant difference in all three dimensions of schizotypy experienced between at least two groups (see Table 5). Black and Latinx participants experienced significantly higher positive schizotypy than Asian participants (*p* < 0.001). Black and Latinx participants experienced more negative schizotypy than Asian participants (*p* = 0.006 and 0.003, respectively). Finally, Latinx students experienced more disorganized schizotypy than Black and Asian students (*p* = 0.003 and <0.001, respectively). We also noted gender differences in multidimensional schizotypy. Gender-minority participants experienced significantly more positive schizotypy than men (*p* = 0.042). There were no noted gender disparities in negative schizotypy. Women experienced significantly more disorganized schizotypy than men (*p* = 0.002). Gender-minority students experienced the most items in all three dimensions of schizotypy; however, due to our limited sample size for this population, the results did not reach significance.

### 3.5. Correlations

Our hypothesis that trauma was positively correlated with positive, but not negative or disorganized, schizotypy was partially supported (see Table 6). Trauma was significantly positively correlated with all dimensions of multidimensional schizotypy. Trauma exhibited medium correlations with positive schizotypy (*r* = 0.43, 95% confidence interval (CI) = [0.37, 0.48], and *p* < 0.001; negative schizotypy, *r* = 0.33, 95% CI = [0.27, 0.39], and *p* < 0.001). Additionally, there was a large correlation between trauma and disorganized schizotypy (*r* = 0.53, 95% CI = [0.46, 0.58], and *p* < 0.001).

Our hypothesis that racial discrimination was positively correlated with positive and disorganized, but not negative, schizotypy was partially supported (see Table 6). Racial discrimination exhibited small correlations with positive (*r* = 0.24, 95% CI = [0.17, 0.31], and *p* < 0.001), negative (*r* = 0.09, 95% CI = [0.02 to 0.16], and *p* = 0.01), and disorganized schizotypy (*r* = 0.08, 95% CI = [0.01, 0.15], and *p* = 0.02).

### 3.6. Moderation Analysis

Racial discrimination did not moderate the relationship between trauma and any dimension of multidimensional schizotypy (see Table 7). For positive schizotypy, trauma and racial discrimination accounted for 18% of the variance, but the interaction failed to reach a level of significance (β = 0.04, SE = 0.07, and *p* = 0.54). For negative schizotypy, racial discrimination, trauma, and their interaction accounted for 8% of the variance, but the interaction failed to reach a level of significance (β = 0.08, SE = 0.07, and *p* = 0.21). Finally, for disorganized schizotypy, racial discrimination, trauma, and their interaction accounted for 23% of the variance in the model. Notably, the interaction for disorganized schizotypy was the closest to approaching significance (β = 0.12, SE = 0.07, and *p* = 0.11). 

## 4. Discussion

The current study has expanded our understanding of the relationships between trauma, racial discrimination, and multidimensional schizotypy among college students. Several interesting findings emerged. First, our team observed numerous gender and racial differences in trauma and racial discrimination. Second, we found that trauma was associated with all three dimensions of schizotypy; however, racial discrimination was only associated with positive and disorganized schizotypy. Finally, contrary to our hypothesis, racial discrimination did not moderate the relationship between trauma and schizotypy. 

We observed numerous gender and racial differences in trauma and racial discrimination. Racial and gender disparities in potentially traumatic experiences were predominantly related to sexual trauma, the unexpected loss of loved ones, and familial separation. Notably, these disparities were all in forms of interpersonal trauma, which can be especially important for adverse mental health outcomes [39]. Additionally, the unexpected loss of loved ones and familial separation may increase feelings of loneliness, which has documented associations with the psychosis spectrum [56,57,58]. Together, these findings emphasize the importance of looking at discrepancies in trauma types across demographic groups. Our team also observed demographic differences in the frequency of racial discrimination. Black participants consistently experienced the greatest levels of racial discrimination in all nine domains measured by the Major Lifetime Discrimination Scale. Given these findings, researchers must conduct analyses on racial discrimination by racial groups. Conducting analyses on People of Color as a unit may ignore important between- and within-group differences. We also must consider the role of the setting as a moderator of the relationship between racial discrimination and adverse outcomes. Black and Asian participants experienced similar levels of racial discrimination in school settings, suggesting that this may be an important site for research and intervention for college students.

Contrary to our hypothesis, trauma was associated with all three dimensions of schizotypy. Trauma exhibited medium correlations with positive schizotypy and negative schizotypy and a large correlation with disorganized schizotypy. Although our findings support previous research on the relationship between trauma and the positive symptoms of psychosis [27,28], our results contradict the literature on trauma and negative and disorganized schizotypy [28,29,33]. However, much of the literature on trauma and psychosis examines childhood trauma [29]. Our findings suggest that a life-course perspective on trauma may be beneficial. Lysaker and LaRocco [33] also used a lifetime measure of trauma, the Trauma Assessment for Adults (TAA)—Brief Revised Version. One limitation of the TAA—Brief Revised Version is that it only asks about war exposure, accidents, natural disasters, and serious illness. The lack of interpersonal trauma data may explain why they did not find that traumatic experiences were associated with disorganized symptoms. Additionally, our field could benefit from a symptom dimension analysis of the PCL-5. For instance, it is plausible that participants high in negative alterations in mood and cognition may also experience more negative and disorganized schizotypy items. In summary, we need to use a lifespan approach to trauma and examine symptom types in order to understand how trauma may be associated with schizotypy.

Our findings partially supported our hypothesis that racial discrimination was positively correlated with positive and disorganized, but not negative, schizotypy. Racial discrimination exhibited small correlations with positive, negative, and disorganized schizotypy. These findings align with epidemiological studies suggesting that racial discrimination is associated with an increased experience of unusual thinking, delusions, disorganization, hallucinations, and paranoia among Students and Adults of Color [18,22]. Additionally, numerous studies have identified an association between racial discrimination and attenuated positive psychotic symptoms [18,59]. The association between racial discrimination and negative schizotypy is a novel finding. Previous research suggests that both racial discrimination and negative schizotypy are associated with a decreased positive effect [39,43,60]. This begs the question if the association between racial discrimination and negative schizotypy is driven by a reduced positive effect. Future studies must examine if a positive effect moderates the relationship between racial discrimination and negative schizotypy. The relationship between racial discrimination and disorganized schizotypy is consistent with previous findings that exposure to racial discrimination depletes cognitive resources [61,62]. 

Racial discrimination did not moderate the relationship between trauma and any dimension of multidimensional schizotypy. Notably, the models, including our variables of interest and their interactions, accounted for 8 to 23% of the variance in multidimensional schizotypy. The greatest amount of variance accounted for was in disorganized schizotypy. Similarly, the interaction for disorganized schizotypy was the closest to approaching significance and, after probing the interaction, we found that at high levels of racial discrimination, the association between trauma and disorganized schizotypy was stronger than low or moderate levels. Our model suggests that there was a large amount of overlapping variance in trauma and racial discrimination observed. It is plausible that racial discrimination and trauma have an additive rather than multiplicative effect as environments that are traumatic may perpetuate high levels of racial discrimination and vice versa. However, before testing alternate models, these findings must be examined in an older community sample. Previous discrimination research has found that older adults report more racial discrimination than middle-aged adults, suggesting that a lifespan approach must be taken [63].

Although this study has added to our understanding of the association between trauma, racial discrimination, and multidimensional schizotypy, this study was not without limitations. As stated earlier, the results should be cautiously interpreted, given that our findings are limited to a predominantly emerging adult sample. Furthermore, our sample reported low levels of discrimination, traumatic stress, and schizotypy. Future studies should supplement university samples with community and clinical samples. Additionally, while our sample included data from six different universities, data from individuals attending schools in the Western and Eastern United States would have been beneficial. Also, we did not recruit many gender-minority Students of Color. Our results suggest that these students experience heightened schizotypy. Future studies must intentionally sample these studies to understand the differential risk of experiencing psychosis. Furthermore, item-level correlations and regressions of the MLDS, LEC-5, PCL-5, and MSS would have furthered our understanding of the relationship between our variables of interest. Despite these limitations, this study examined a novel model of risk in symptoms of psychosis and has helped our field move closer to understanding between- and within-group differences by examining the role of gender, race, and ethnicity.

## 5. Conclusions

In summary, this study has extended the literature by examining how racial and gender differences in experiences of trauma and racial discrimination may impact the prevalence of schizotypy among People of Color. Previous findings that racial discrimination and trauma have a significant positive correlation with positive schizotypy were replicated. Together, the racial discrimination and schizotypy literature suggests that racial discrimination may partially account for the heightened prevalence of schizotypy among People of Color. System-level interventions funded at state and federal levels are crucial to decrease the disparities in psychosis risk. Additionally, we found that trauma and racial discrimination were associated with all dimensions of multidimensional schizotypy, with the association between trauma and disorganized schizotypy being the strongest. These findings suggest that there should be increased screening and intervention for people who experience a history of interpersonal trauma and high schizotypy. Racial discrimination did not moderate the relationship between trauma and multidimensional schizotypy, but approached significance for disorganized schizotypy. Additionally, although the racial discrimination and trauma literature has largely focused on the positive symptoms of psychosis, disorganized schizotypy showed the most robust associations and may be a critical site for intervention. Future research must incorporate lifespan and profile analyses among more symptomatic samples to understand how previous life experiences increase disorganized schizotypy. If the literature continues to suggest that racial discrimination and trauma serve as risk factors for the severity of schizotypy and the eventual development of schizophrenia-spectrum disorders, we must develop culturally syntonic interventions for People of Color experiencing symptoms of psychosis to aid their recovery. 

## Figures and Tables

**Table 1:** Demographic data.

Variable	Range	α	M (SD)
Age	18–43	-	19.24 (2.00)
PCL-5	0–80	0.95	20.85 (17.72)
MLDS	0–25	0.81	4.52 (4.68)
MSS Positive	0–26	0.89	5.59 (5.30)
MSS Negative	0–21	0.86	4.24 (4.54)
MSS Disorganized	0–25	0.94	6.32 (6.90)

Notes. α refers to Cronbach’s alpha. M: mean; SD: standard deviation; PCL-5: Post-Traumatic Stress Disorder (PTSD) Checklist for Diagnostic and Statistical Manual of Mental Disorders Fifth Edition (DSM-5); MLDS: Major Lifetime Discrimination Scale; MSS: Multidimensional Schizotypy Scale.

**Table 2:** Gender differences in trauma symptoms.

	Men (*n* = 192)	Women (*n* = 562)	GM (*n* = 14)	*p*-Value	Group Differences
Natural disaster	2.28 (1.11)	2.44 (1.20)	2.21 (0.70)	0.221	None
Fire or explosion	2.16 (1.17)	2.03 (1.09)	2.14 (0.77)	0.330	None
Transportation accident	3.15 (0.98)	2.98 (1.19)	2.64 (1.22)	0.098	None
Serious accident	2.42 (1.35)	2.07 (1.36)	2.21 (1.42)	0.010 *	None
Toxic exposure	1.41 (1.13)	1.35 (1.11)	1.43 (1.02)	0.818	None
Physical assault	2.45 (1.42)	2.44 (1.39)	3.00 (1.41)	0.334	None
Weapon assault	1.46 (1.13)	1.48 (1.06)	1.57 (1.34)	0.932	None
Sexual assault	1.49 (1.16)	2.12 (1.39)	2.57 (1.45)	<0.001 **	2, 3 > 1
Unwanted sexual experience	1.85 (1.41)	2.51 (1.51)	2.93 (1.69)	<0.001 **	2, 3 > 1
Combat or war zone	1.14 (1.04)	1.11 (1.00)	1.14 (1.01)	0.320	None
Captivity	1.04 (1.00)	1.04 (1.00)	1.07 (1.00)	0.994	None
Illness or injury	1.92 (1.27)	1.89 (1.34)	1.64 (1.39)	0.744	None
Severe human suffering	1.68 (1.18)	1.42 (1.17)	1.50 (1.29)	0.032 *	1 > 2
Sudden violent death	1.40 (1.00)	1.50 (1.02)	1.29 (1.00)	0.348	None
Sudden death	1.82 (1.11)	2.04 (1.15)	1.64 (1.15)	0.043 *	None
Serious harm to others	0.80 (1.23)	0.52 (1.01)	0.43 (0.76)	0.007 *	1 > 2
Other stressful event	2.77 (1.53)	2.81 (1.54)	2.71 (1.59)	0.919	None
Prolonged separation	1.30 (1.49)	1.28 (1.60)	2.29 (1.90)	0.062	3 > 2

Note. Data are presented as mean (standard deviation) for all variables. “Group Differences” is a synopsis of mean differences in traumatic events across genders. GM: gender-minority. * *p* < 0.05; ** *p* < 0.001.

**Table 3:** Racial and ethnic differences in trauma symptoms.

	Black (*n* = 191)	Latinx (*n* = 358)	Asian (*n* = 221)	*p*-Value	Group Differences
Natural disaster	2.42 (1.18)	2.34 (1.19)	2.48 (1.14)	0.330	None
Fire or explosion	2.14 (1.19)	2.04 (1.07)	2.05 (0.96)	0.569	None
Transportation accident	3.06 (1.12)	3.02 (1.20)	2.95 (1.08)	0.624	None
Serious accident	2.12 (1.45)	2.18 (1.34)	2.17 (1.30)	0.847	None
Toxic exposure	1.27 (1.16)	1.36 (1.10)	1.48 (1.12)	0.155	None
Physical assault	2.52 (1.49)	2.53 (1.34)	2.29 (1.40)	0.108	None
Weapon assault	1.55 (1.15)	1.52 (1.10)	1.35 (0.99)	0.109	None
Sexual assault	1.99 (1.46)	2.12 (1.36)	1.71 (1.28)	0.002 *	2 > 3
Unwanted sexual experience	2.44 (1.53)	2.54 (1.49)	1.96 (1.50)	<0.001 **	1, 2 > 3
Combat or war zone	1.14 (1.03)	1.17 (1.02)	1.10 (0.98)	0.703	None
Captivity	1.05 (1.04)	1.07 (0.99)	0.99 (0.99)	0.630	None
Illness or injury	2.03 (1.38)	1.90 (1.29)	1.76 (1.31)	0.105	None
Severe human suffering	1.39 (1.18)	1.58 (1.17)	1.43 (1.17)	0.144	None
Sudden violent death	1.58 (0.98)	1.47 (1.02)	1.38 (1.03)	0.121	None
Sudden death	2.20 (1.06)	1.97 (1.16)	1.80 (1.15)	0.002 *	1 > 3
Serious harm to others	0.74 (1.20)	0.55 (1.05)	0.51 (0.98)	0.059	None
Other stressful event	2.90 (1.57)	2.76 (1.54)	2.77 (1.51)	0.578	None
Prolonged separation	1.29 (1.64)	1.34 (1.56)	1.24 (1.56)	0.797	None

Note. Data are presented as mean (standard deviation) for all variables. “Group Differences” is a synopsis of mean differences in traumatic events across racial and ethnic groups. * *p* < 0.05; ** *p* < 0.001.

**Table 4:** Racial and ethnic differences in racial discrimination.

	Black (*n* = 191)	Latinx (*n* = 358)	Asian (*n* = 221)	*p*-Value	Group Differences
At school	1.55 (1.17)	0.92 (1.07)	1.48 (1.14)	<0.001 **	1 > 2
Getting a job	0.51 (0.89)	0.17 (0.50)	0.17 (0.54)	<0.001 **	1 > 2, 3
At work	0.79 (1.06)	0.45 (0.85)	0.46 (0.89)	<0.001 **	1 > 2, 3
Getting housing	0.17 (0.59)	0.07 (0.34)	0.05 (0.27)	0.003 *	1 > 2, 3
Getting medical care	0.35 (0.73)	0.16 (0.50)	0.12 (0.43)	<0.001 **	1 > 2, 3
Getting service in store or restaurant	1.34 (1.12)	0.60 (0.92)	0.66 (0.91)	<0.001 **	1 > 2, 3
Getting credit or bank loans	0.19 (0.60)	0.08 (0.37)	0.06 (0.32)	0.004 *	1 > 2, 3
In public	1.34 (1.16)	0.77 (1.05)	1.07 (1.13)	<0.001 **	1 > 3 > 2
From police or courts	0.58 (0.95)	0.27 (0.65)	0.18 (0.49)	<0.001 **	1 > 2, 3

Note. Data are presented as mean (standard deviation) for all variables. “Group Differences” is a synopsis of mean differences in racial discrimination across racial and ethnic groups. * *p* < 0.05; ** *p* < 0.001.

**Table 5:** Gender, racial, and ethnic differences in multidimensional schizotypy.

	Men (*n* = 192)	Women (*n* = 562)	GM (*n* = 14)	*p*-Value	Group Differences	Black (*n* = 191)	Latinx (*n* = 358)	Asian (*n* = 221)	*p*-Value	Group Differences
MSS Positive	4.96 (4.97)	5.75 (5.33)	8.50 (7.42)	0.042 *	3 > 1	6.52 (5.42)	6.03 (5.43)	4.09 (4.66)	<0.001 **	1, 2 > 3
MSS Negative	3.93 (4.14)	4.31 (4.66)	5.21 (5.12)	0.43	None	4.67 (4.59)	4.59 (4.71)	3.30 (4.08)	<0.001 **	1, 2 > 3
MSS Disorganized	4.82 (6.18)	6.79 (7.03)	8.93 (8.34)	0.002 *	2 > 1	5.65 (6.19)	7.63 (7.29)	4.78 (6.45)	<0.001 **	2 > 1, 3

Note. Data are presented as mean (standard deviation) for all variables. *n*: Number of participants; GM: gender-minority; MSS: Multidimensional Schizotypy Scale. * *p* < 0.05; ** *p* < 0.001.

**Table 6:** Intercorrelations among study variables (*n* = 770).

Variable	MSS Positive	MSS Negative	MSS Disorganized
PCL-5	0.43 ** [0.37 to 0.48]	0.33 ** [0.27, 0.39]	0.53 ** [0.46, 0.58]
MLDS	0.24 ** [0.17, 0.31]	0.09 ** [0.02 to 0.16]	0.08 ** [0.01 to 0.15]

Notes. Two-tailed Pearson correlations with 95% confidence intervals in brackets. PCL-5: Post-traumatic Stress Disorder (PTSD) Checklist for Diagnostic and Statistical Manual of Mental Disorders Fifth Edition (DSM-5); MLDS: Major Lifetime Discrimination Scale; MSS: Multidimensional Schizotypy Scale. Medium effect sizes in bold; large effect sizes in bold and italics. ** *p* < 0.001.

**Table 7:** Racial discrimination as a moderating variable in relationships between trauma and schizotypal traits (*n* = 770).

	Positive				Negative				Disorganized			
	*R* ^2^	*β*	SE *B*	*p*	*R* ^2^	*β*	SE *B*	*p*	*R* ^2^	*β*	SE *B*	*p*
Overall Variance	0.18				0.08				0.23			
Trauma		0.27	0.04	<0.001 **		0.17	0.04	<0.001 **		0.36	0.05	<0.001 **
RacialDisc		0.08	0.08	0.33		−0.09	0.08	0.30		−0.15	0.09	0.11
Trauma X RacialDisc		0.04	0.07	0.54		0.08	0.07	0.21		0.12	0.07	0.11

Notes. *R*^2^: coefficient of determination; *β*: unstandardized beta; SE *B*: standard error for unstandardized beta. ** *p* < 0.001.

## Data Availability

The data presented in this study are available on request from the corresponding author.
